# Extra Supplement.—The Nursing Mirror

**Published:** 1894-03-24

**Authors:** 


"The Hospital" Nursing Mirror.

Being the Extra Nursing Supplement of "The Hospital" Newspaper.

[Contributions for this Supplement should be addressed to the Editor, The Hospital, 428, Strand, London, W.C., and should have the word "Nursing" plainly written in left-hand top corner of the envelope.]

### News from the Nursing World.

### HAMPSTEAD HOSPITAL.

The Hampstead Home Hospital has decided to change its name to the simpler title of Hampstead Hospital, and its general wards will in future be free for the use of poor patients living in Hampstead, Highgate, Hendon, and Kentish Town, who obtain a subscriber's letter. Some special wards will still be reserved for paying patients.

### A WARNING TO NURSES.

We constantly hear complaints of lost testimonials from Nurses writing to tell of their having applied for posts at private institutions and enclosing by request their photographs and original testimonials, and when they fail to obtain the advertised vacancy they seem often to experience great difficulty in regaining the papers which are such extremely important possessions. We can only advise, as we have always, that nurses should never send their original testimonials and certificates to any stranger; they can get copies typed or printed at a small expense and retain the originals in their own safe custody to be shown when needful, but not to be lightly parted with.

### DISTRICT NURSING AT RICHMOND.

The Richmond branch of the St. John Ambulance Association is said to be the first guild which organised voluntary amateur nursing of the sick poor. The associates have been most energetic in visiting and looking after their patients, and they have now decided to try and secure a fully-trained district nurse to work in connection with the guild. All who were present at the annual meeting seem fully to realise the boon it will be for the more serious cases to have the services of a qualified nurse, whilst the lady associates still propose to devote themselves to the care of chronic and slight ailments. It is somewhat unusual nowadays to find so large a town as Richmond possessing no trained district nurses of its own, and the associates of the St. John Ambulance Guild are to be congratulated for having drawn public attention to the existing need.

### ROYAL NATIONAL PENSION FUND FOR NURSES.

The following criticism of the report and accounts for the year ending December 31st, 1893, is taken from the money article of the *Times*: The balance-sheet of the Royal National Pension Fund for Nurses shows that at the end of last year the assets were £167,080, invested in safe securities. Chief among the liabilities is the Annuity Fund of £111,535; the Donation Bonus Fund is £34,793; the First Thousand Nurses' Tontine Fund is £5,952; and the J. S. Morgan Benevolent Fund amounts to £11,061. A quinquennial valuation has been made by Mr. George King, the honorary actuary, who reports that the sum available for distribution amongst the holders of annuity contracts out of the donation bonus fund account was £4,028, which, with £5,000 of the capital of the fund and accumulated interest, has been turned into annuities corresponding to those already held by those concerned. Mr. King congratulates the Council and the members on "the very prosperous condition of the Pension Fund" and on "the large increases which will be possible in the annuities of those nurses who do not withdraw from membership." It is certainly satisfactory to find that so excellent an institution is prospering financially.

### "ON DUTY NIGHT AND DAY."

Under this heading The Hospital of February 24th called attention to the overworking of the nurses at Cheltenham Workhouse Infirmary, supervision of serious cases being actually demanded during the night from the day nurses. At a recent board meeting, reported by the *Cheltenham Examiner*, Mr. Feeney called attention to the paragraph in question, and frankly mentioned that he had since found the average of patients in bed was exactly twice as great as the number he had given in the report quoted by The Hospital. He also "hoped this fact would arouse public opinion, and do something to remove the scandal." Doubtless this wish will shortly be realised, although the Chairman may consider that the Cheltenham patients are already "quite as well attended to as anywhere."

### GIRL-NURSES AGAIN.

"You must send him to the Small-pox Hospital at once," says the doctor, called in to see a case in a small private house, and his verdict is generally acquiesced in by the patient's friends. In other illnesses the necessity for such strict isolation may be disputed, but small-pox is dreaded by most English folks. When the unlucky victim becomes an inmate of the infectious hospital, he is as completely lost sight of by his family as if he were undergoing a short period of penal servitude, therefore it surely becomes the duty of everyone to ensure that those who are voluntarily incarcerated for the public good, should be well tended. Sick men and women are almost as helpless as little children, and need as great care. The recent tragedy at the Birmingham Small-pox Hospital has, according to the local press, revealed what it is to be hoped is a unique system of nursing, for the so-called "nurse," placed alone on night duty with thirty-eight patients, one of whom was violently delirious, is now reported to be a probationer only eighteen years of age. Some half-dozen of children, including a baby, are said to have been among the responsibilities laid upon this young girl. Isolation hospitals will certainly be looked upon with considerable distrust if the comfort, nay, the very lives of people suffering from serious diseases, are left to such casual supervision.

### ROYAL BRITISH NURSES' ASSOCIATION.

A correspondent wishes to know why the Royal British Nurses' Association has abandoned the fine suite of rooms in the house of the late Sir Morell Mackenzie. We understand that the fine suite of rooms has abandoned the Association, seeing that the trustees of the ground landlord refused to sanction the proposed arrangement.

### BRISTOL NURSES' TRAINING INSTITUTE.

The annual meeting of the Bristol Nurses' Training Institution and Home took place on 13th inst., and a good report was submitted by Miss O'Brien. After the adoption of the report the election of the Committee took place, Mrs. Watson Williams being appointed to fill a vacancy.

### AN URGENT APPEAL.

If the charges brought by the nurse at Newton Abbot Workhouse are true ones, she deserves the thanks of the community for her courage in making them public. The Local Government Board has been urged to institute an inquiry into the serious evils which are alleged to exist, and which include "gross immorality amongst the inmates" and an appalling condition of dirt. At the last meeting, as reported by the local press, it was decided that steps must be taken to prove the truth or falsehood of these grave charges before the departure of the nurse, whose engagement in the institution is now drawing to a conclusion.

### THE SENIOR NURSE.

The Guardians at Ballycastle Union are desirous of increasing their nursing staff, which proves that they have the true interests of the inmates at heart. It is, however, a great pity that some of these kindly gentlemen have not a little preliminary knowledge of the methods followed in other institutions where trained nursing is satisfactorily established. The Ballycastle Guardians appear nervous lest the present nurse whom they have engaged for three months should run the risk of clashing with the "trained probationer nurse" they propose to get, so it is evident they do not realise that the senior and most experienced nurse must naturally take the lead. The interests of both women should centre in the patients, for whose benefit they are presumably to work, and it rests with the Guardians to see that matters are properly organised from the outset. Then there will be no question afterwards of any disastrously divided authority.

### QUEEN'S NURSES AT ABERDEEN.

The appreciation accorded to the work of the Queen's nurses at Aberdeen is so great that it will surely eventually secure increased financial support for the District Nursing Association. The staff now consists of a superintendent and three nurses, and the unsectarian character of the Institute received as hearty approval at the annual meeting as did its valuable work.

### NURSING AT AYR.

The nineteenth annual report of the Ayr Sick Poor Nursing Association recently issued is a noble record of good work. The Committee are very anxious for increased subscriptions to justify them in securing a second nurse. The Association receives staunch support from all the institutions in the district, but larger financial aid is greatly desired. Nurse M'Callum is still highly valued by those amongst whom she works, and the ladies of the neighbourhood supplement her labours by many gifts for the use of the sick and poor of Ayr.

### NUNS AT CHICAGO.

The unalluring title of "The Pest-house" is borne by the building at Chicago where the victims of small-pox are placed and tended. It is under the charge of the Sisters of the Order of Poor Handmaids of Jesus, and they have been unremitting in their attentions to the patients, some one hundred and twenty in number, who have passed through their hands. A Sister, named Mary Albina Hummert, herself fell a prey to the disease, and died there after seven days' illness. She was one of the most energetic and valued, as well as one of the oldest, members of the nursing staff.

### ANOTHER NEW NURSES' HOME.

The nurses connected with the German Hospital and Dispensary in New York have now had their new Home formally opened. The board of trustees gave a reception on the occasion, and the new building was inspected by many visitors. It is a fireproof, five-storey structure, built of red brick and well lighted by windows on all four sides. Of course all modern improvements, both of fittings and structures, have a place in the new Home.

### A VALUED ARMY NURSE.

The death, at Washington, at the age of 73, of Mary Morris Husband, on the 3rd inst., will be deeply regretted by many an American soldier who owes his life, humanly speaking, to her good offices. Mrs. Husband's two sons having joined the Union forces at the breaking out of the war, she employed herself for some time in nursing in the hospitals at Philadelphia. When the news reached her that one of her sons, who had gone to the front, was lying seriously ill, unencumbered by any luggage beyond a hand satchel, she immediately set off, and had the happiness of nursing him back to health. She also found herself able to do a great deal for other sick soldiers, and her services were enormously valued, the men bestowing on her the affectionate title of "Mother Husband." From this time she threw her whole energies into the service of the sick and wounded, was twice under fire without sustaining any injury, and was with the field hospitals of the army of the Potomac, and at Gettysburg suffered from a dangerous attack of fever.

### A FAITHFUL NURSE.

"She served the sick faithfully for twelve years, and died in harness" is surely as fine an epitaph as any nurse could wish for. Of Teresa Christian, who died at 33, Beaumont Street, after a very short illness, Miss Meyrick says that and a great deal more, for she was an admirable nurse and a true friend, and her loss is deeply felt by many who respected her. Miss Meyrick's Nursing Hostel, of which we gave a notice on its opening a year and a half ago, has proved a most comfortable home to a number of patients since then, and we wish it continued success.

### SHORT ITEMS.

The annual meeting of the Kingston Nursing Association was recently held in the town, and a satisfactory report was presented. In September a fourth nurse was added to the staff, and her services have been available for paying patients.—The Llanelly Guardians have appointed Miss Mary Colbart nurse of the Union workhouse at £30 per annum, and are advertising for an untrained assistant nurse at £15.—The Committee of the Retford Dispensary and Cottage Hospital have received a promise of £200 towards the fund for securing a trained nurse to attend patients in their own homes.—The work done by the Bolton District Nursing Association during the last year has been excellent and extensive. A hundred and five cases of typhoid and many patients with pneumonia and other serious diseases have been satisfactorily tended by the six nurses now attached to the institution.—Finedon is following the good example of Rushendon and Wellingborough in the matter of a trained district nurse for the sick poor.—The *Nurses' Journal* reports the expulsion of Nurse M. E. MacGregor from membership of the R.B.N.A. on 24 January by 37 votes out of 41 present at the Council.

### On General Nursing.

By ROWLAND HUMPHREYS, M.R.C.S., L.R.C.P.Lond.

### IV.—SCARLET FEVER (*Continued*).

### OTHER SYMPTOMS.

*The Tongue.*—This is at first coated, as in all febrile complaints; soon red papillæ begin to show through the fur, and about the same time as the skin begins to peel the tongue does the same. This, as has been pointed out, is a result of the developmental origin of these structures, both of them having the same origin. After peeling, the papillæ remain for some time very prominent, giving the tongue much the same appearance as that of a cat. In bad cases it remains dry or ulcerates.

*The Pulse*, at first very rapid, becomes gradually slower, and becomes normal about the end of the second week. It becomes more rapid if any local inflammatory lesion threatens, and slower or more often irregular if inflammation of the kidneys has set in. The last-mentioned complication is frequently so insidious in its onset that this symptom becomes a very valuable one.

*Delirium.*—It has been noticed (Gresswell) that this symptom is most common in males over ten. If found with high temperature and quick pulse it is indicative of the malignant form of the fever.

*Vomiting*, as an early symptom, is always suggestive of the complaint of which we are treating; at a later stage it shows meningitis, especially if combined with mental symptoms, which get worse towards night, and with a rise in the temperature.

### COMPLICATIONS.

A long list might be made of the complications which attend scarlet fever, but we will content ourselves with mentioning a few of the more prominent and dangerous ones.

*Nephritis*, or inflammation of the kidneys, takes first rank among the dangers peculiar to an attack of this complaint. There is a distinct tendency to it at the very first onset of the fever, though this is then more of the nature of a temporary suppression due to the overgrowth of the epithelium, or membrane, which covers those parts of the kidney which serve to drain off the watery part of the urine from the blood. The other period, when any attack is serious, occurs in the second or third week, but it may come on at any period of the complaint while the skin is peeling.

The symptoms are either (1) those of fever, with partial or total suppression of urine, what flows being dark and contains blood, and "casts," formed by epithelial cells, which have taken the form of the tube in the kidney from which they have come. The urine passed is of high specific gravity, but the amount of urea which passes daily from the body is less than it should be, and other waste materials, which should pass off by the same route but are also retained, cause the symptoms of uræmia. Dropsy sets in early, and if not quickly relieved, the injury to the kidney gets beyond repair, and terminates sooner or later in the death of the patient.

The other form (2) comes on without rise of temperature, and is shown by the swelling of face, extremities, and body with albuminuria. The low specific gravity of the urine shows that the amount of the solids which are held dissolved in the urine is low, and the colour is pale. It has been noticed that the colour of the urine in this last form of the complaint becomes gradually paler and paler for some time previous to the appearance of albumen, and though this requires careful observation, yet the advent of pale urine in these cases should not be passed over as of no consequence.

The renal disease usually subsides under appropriate treatment. It may, in almost all cases, be avoided by treating the case from the first appearance of the rash as if it were one of acute Bright, restricting the animal diet, and seeing that the skin acts well, and the bowels are regularly moved. The kidney "is not an organ which recovers quickly," so that the case has to be watched and guarded for a long time after an attack of nephritis; otherwise the case may become, as it too often does, one of "Chronic Bright," ending life in the course of a few months.

The best and simplest test for albumen lies in boiling the suspected urine in a test tube, and then adding a few drops of acetic acid; if a cloud then appears, or if previously present still remains albumen is present. The amount of albumen passed from day to day may be compared by pouring the urine after boiling into a graduated measure, and allowing it to settle for twenty-four hours.

The next complication, of which it is necessary to take notice, is that of acute arthritis, often called acute rheumatism, which in this disease is due, probably, to the septic nature of the infection. The author has seen the joint affection precede the rash, but this is unusual, and it does not usually come on till the second week. It is treated in just the same way, and has similar symptons and dangers to rheumatic fever.

Another complication is where the throat takes on a severe form of ulceration. This often results in inflammation of the glands of the neck, followed by suppuration. It may also extend up the eustachian tube to the ear, causing perforation of the drum, with more or less permanent deafness; or it may cause an abscess to form in the mastoid process of the temporal bone, or may cause necrosis of the very thin layer of bone which alone separates the tympanic cavity from that of the brain, and may then set up cerebral abscess. In these cases the child first complains of earache, worse at night; or if too young to explain its troubles, keeps throwing its head from side to side and crying continuously. The temperature rises, there may be a rigor or convulsion, and discharge usually appear from the ear, and the glands of the neck below the ear become inflamed. With these symptons measures for the relief of the patient cannot be taken too quickly. The great thing is to prevent the extension of the disease from throat to ear, and if discharge has appeared, to see that the ear is kept quite sweet. The former may be effected by constant attention, in bad cases, to the ulceration, douching the nose out with antiseptic applications two or three times a day, and spraying or painting the inside of the throat with such remedies as the chlorinated solution of soda diluted one part to thirty of water, or boracic acid and glycerine, or solution of the perchloride of mercury, or other antiseptic. The ear may be douched out with similar solutions, a little boracic acid in powder being blown in after the douching, and, as far as possible, drying it. It is then plugged with a piece of antiseptic wool. The earache may be relieved by a drop or two of a weak solution of atropine put into the ear.

In bad throat cases it is often very difficult to get the patient to swallow, and then it is best to feed through the nose. This is really a very simple proceeding, and affords wonderful comfort to the patient by preventing the pain caused by swallowing. A piece of baby's bottle tubing six inches long attached to a 3 oz. glass funnel, is passed along the floor of the nostril (which corresponds to the roof of the mouth) in a backward direction, allowing it to find its own way down as far as it will go. If the patient begins to cough it must be withdrawn for an inch, and the rest allowed to be done by swallowing. It is very easy to regulate the flow by bending or pinching the tube, and time should frequently be allowed for breathing. It is best to lay the patient right across the bed, with the head close to the edge next the feeder, and then all movements can easily be restrained. The tube can also be used for feeding fractious children by passing it inside the cheek as far as the back of the mouth. Beef-tea, milk, eggs, and medicines can thus be administered.

### Lectures at St. George's Hospital.

### THE ANTISEPTIC SYSTEM.

Mr. Dent has been continuing his course of lectures to nurses at St. George's Hospital. Elementary anatomy and physiology have been dealt with, and on Tuesday, March 13th, was given the first of three lectures on "The Antiseptic System."

"It is essential for every nurse to understand the principles on which the antiseptic system of wound treatment is based," and that the system may be efficiently carried out it is necessary that all who have to do with the patient shall believe thoroughly in it. Mr. Dent spoke of the assertion that "lives are saved in proportion to the asepticity of the wound," as a strong but perfectly true one, and of the fact that with the antiseptic system everyone concerned in the care of a patient has an equal share in the responsibility. There is, perhaps, this drawback to the modern system of antiseptic surgery, that whereas in old days the surgeon had the case pretty much in his own hands, and if successful the credit was much more his than anyone else's, and *vice versa*, with the present treatment all who are in any way in contact with the case the surgeon, the house surgeon, the nurses, the maker of the instruments, and the instrument cleaner share the responsibility. It is the case of a multitude of cooks, and it follows that sometimes the broth is spoilt. But statistics show an enormous saving of life by the modern form of treatment. In Sir Joseph Lister's practice at the Edinburgh Infirmary cases of amputation were tabulated, and taking three years' experience of a good many years ago, a mortality is given of 45 per cent., nearly one in every two cases, while under the antiseptic system in the same wards, in the same place, under the same surgeon, with nothing else changed, and with the same class of patients, the mortality is reduced to 15 per cent., one in six, a third of the old mortality. At St. George's Hospital cases of amputation have been tabulated since 1852, and show a corresponding decrease in mortality. A stronger proof still of the saving of lives is found in the fact that at St. George's, in former years, cases of compound fractures were tabulated, many of these patients dying of pyæmia, erysipelas, and other preventible diseases, while many more lost their limbs. These tables dated from about 1854, but since the days of antiseptics these books have been given up altogether, for the reason that there is practically now no mortality from these cases at all.

Mr. Dent then proceeded to describe the process of fermentation and its results, instancing the effect of introducing a ferment such as yeast to grape juice, as is done in wine making, and comparing the changes which then take place to those which go on in the human body in the case of diseases which are known as "zymotic" diseases—*i.e.*, fermentative diseases, the process being very akin to fermentation, and it being believed that the changes which take place in wounds may be similar to those which take place in the grape juice, rise of temperature, &c., and more especially infinite multiplication of the ferment. Putrefaction, too, both in vegetable matter and in wounds is a further stage of fermentation, with the giving off of bad smell. If, therefore, fermentation is prevented, so also is putrefaction, the object of the antiseptic system being essentially to prevent these conditions.

Mr. Dent showed some cultures of various bacteria, and described the multiplication of the cells which were known by the shape, size, and method of grouping together, going on to explain that while in the blood of a healthy person no such organisms are found, in a patient suffering from cholera or other disease, if the blood be examined, it is seen to be full everywhere of millions of these little bacilli. Explaining the precise meaning of the terms "antiseptic" and "aseptic," Mr. Dent demonstrated their signification by showing a vessel containing some clear soup, which had been kept good for a fortnight with a cotton wool plug, through which filtered air only was admitted, all injurious particles of dust, &c., being kept out; and an experiment with a lime-light lantern and water of various degrees of purity gave an alarming idea of the properties of the latter. But there are other dangers in London and large towns which are not to be avoided. A man suffering from consumption, going along the street, spits on the pavement. The ferment in the expectoration dries, and is wafted into the air; someone breathes it, and if suitable soil be found then comes in the multiplication of the ferment. But we are all running such risks every day, and are not as a rule a penny the worse, so Mr. Dent comfortably assured his audience, a suitable pabulum for the ferment to multiply in being an essential condition. At the next lecture further aspects of the antiseptic system will come under consideration.

### The Difficulties of Private Nursing.

By One who Knows Them.

"Yes, she was very ill indeed; but, thank God, we were able to do without a hospital nurse," were words I heard spoken by a lady to her friend in a railway carriage the other day. Then followed an exchange of experiences from one and the other as to the delinquencies of the class to which I belonged. Had there been other listeners than myself they would inevitably have thought "hospital nurses" to be a speci s of vampire, invented for the annoyance of all sick people and their friends, rather than women, from whom was expected by the generality of people every virtue under the sun, and also superhuman physical powers of endurance and self-sacrifice. "What, nurse! you require more than four hours' sleep?" said a lady to a friend of mine the other day when the nurse was retiring to rest after a day and a night on duty. That is not an isolated case. Many nurses with good connections amongst doctors, who would be sure of plenty of employment, might work for themselves, taking their own fees, but they prefer to join some institution, and pay a large percentage on all their earnings, that they may shelter themselves behind the rules for rest and exercise these establishments insist upon.

Among the difficulties a private nurse has to contend with is that of convincing the friends that the doctor's orders are to be carried out faithfully and intelligently to the very letter. For instance, I was nursing a very critical case of heart disease, with congestion of the lungs and liver. The directions I received as regards food were, "Very small quantities and very concentrated." I think no one would believe the difficulty I had to prevent unlimited amounts of jellies, all sorts of puddings, and even at times muffins being given. My absence from the room was always the signal for something to be administered, considered by the friends "nice and strengthening," "far better than Valentine's extract or Brand's essence!"

Again, the curious duties some people expect the nurse to perform become often both amusing and perplexing. A friend of mine was sent from London to a house in Yorkshire, and arriving tired and travel-stained, was shown at once into the bed-room of the lady of the house, and was requested by her to lay aside her bonnet and cloak, and go to the kitchen and fetch her hot water, after which she was to brush and lay out for use the clothes of the master of the house. My friend inquired for her patient, and was told she would not be required for nursing that night, but could help wait at table. Needless to say an early train conveyed her back to town next morning. It seems strange that people should be found willing to pay £2 2s. a week for someone to do the work of an upper housemaid.

### The Royal National Pension Fund for Nurses.

THE annual general meeting was held at the offices of the Fund, 8, King Street, Cheapside, on Thursday, March 15th, 1894, at half-past four p.m. Present: Mr. W. H. BURNS (Chairman), Mr. H. C. Burdett, Mr. T. Bryant, F.R.C.S., Mr. A. H. A. Marton, Mr. G. Norman, Mr. G. King, Dr. Potter, Dr. Perry, Mr. E. Rawlings, Mr. Hugh Macpherson, Mr. Charles Coates. Miss Monk, Miss Ross (of Glasgow), Miss Vincent, and several ladies interested in the work were also in attendance.

The SECRETARY read the notice convening the meeting.

The CHAIRMAN nominated Mr. Macpherson and Dr. Potter as scrutineers in connection with the ballot for "eight representatives of the annuitants and policy-holders of the society, who under the articles of association, might be added to the Council."

THE REPORT OF THE COUNCIL.

The Council have the pleasure to submit to the members of the Fund their report with the annexed accounts and balance-sheet prepared in conformity with the statutory requirements, and made up for the period of twelve months ending December 31st, 1893.

Eight hundred and fifty-six policies have been issued from the commencement of the Fund.

INVESTMENTS.—The investments on December 31st, 1893, amounted to £163,689 2s. 1d., as follows: In British Government securities, £20,121 17s. 6d.; in railway and other debentures and debenture stocks, £88,454 1s.; ditto shares (preference and guaranteed), £6,166 6s. 10d; in foreign Government securities, £30,018 16s. 9d.; in Municipal Corporation bonds and stocks, £8,598 9s.; total £153,359 11s. 1d. Investments of the Junius S. Morgan Benevolent Fund: In railway and other debentures, £9,224 10s. 6d.; in foreign Government securities, £1,105 0s. 6d.; total, £10,329 11s. Total invested funds, £163,689 2s. 1d.; cash balances, outstanding interest, loans, &c., £3,391 10s. 9d.; total funds, £167,080 12s. 10d.

PENSION BRANCH.—It is satisfactory to report that the figures continue to show a steady growth in all branches of the business. On the pension side 533 proposals were dealt with during the year, as against 512 in 1892, and 497 pension policies were issued, as against 486 in 1892. From the nature of a nurse's calling and circumstances, experience shows that the Council must expect to have to deal with a number of withdrawals during each year. It is satisfactory to note, however, that the Fund is old enough to prove that former policy-holders, who have surrendered their policies for one reason or another, are re-entering it in increasing numbers each year.

SICKNESS BRANCH.—This branch again shows a material growth in the numbers; 260 proposals were dealt with during the year, as against 182 in 1892; and 216 policies were issued, as against 168 in the previous year. The number of nurses who received sick pay during the year was 129, as against 89 in 1892.

FEDERATION.—The new scheme of federation, approved by the Council in the course of the year, has commended itself for adoption to the authorities of several large institutions. Amongst them may be mentioned Guy's Hospital and Guy's Hospital Nursing Institution, the authorities of which pay the premium for a policy of £11 5s. in favour of each member of their nursing staff who joins the Fund on her own account for a pension of not less than £7 10s. The authorities of the Royal Free Hospital, the Seamen's Hospital, Greenwich, the Metropolitan and National Nursing Association, and the Kent and Canterbury Nursing Institution, have each adopted a scheme of federation suitable to the requirements of their respective institutions.

THE QUINQUENNIAL VALUATION.—In last year's report a supplementary report was promised as soon as the actuary, Mr. George King, F.I.A., F.F.A., had concluded his labours. Owing mainly to the amount of work entailed by the large number of policy-holders, and to the peculiar nature of the business, Mr. George King's report was not received as soon as was anticipated, and the Council determined in consequence to include the results in the report for the year ending December 31st, 1893.

The results of the valuation show that: (1) the sum available for distribution amongst the holders of annuity contracts out of the donation bonus fund account was £4,028 6s. 9d., which sum has been distributed amongst holders of annuity contracts, and applied to increase their annuities; £5,000 of the capital of the donation bonus fund, together with the accumulated interest, has been divided equally amongst those of the first thousand nurses who are still members of the fund. These cash appropriations have been turned into annuities of corresponding kinds to the annuities already held by these nurses. This special fund of £5,000, and the interest thereon, will be always kept separate as a tontine fund, and any annuities profiting by it will be cancelled if the beneficiaires, from any cause, cease to belong to the fund. The values of such cancelled annuities will be applied at each valuation to increase the annuities of those of the first thousand nurses who still remain policy-holders in the fund. (2) The total profits made by the fund under annuity contracts was £727 2s. 6d., and under the sickness contracts, £317 17s. 11d.; but, under the actuary's advice, no part of these profits have been divided amongst the policyholders, and the whole is held in reserve at interest until the next valuation.

On this point the Consulting Actuary reports: "That there should be a surplus, even though small, is highly satisfactory. In the early days of the Pension Fund the expenses of management were of necessity proportionately much heavier than they will be in the future, and until the funds had had time to accumulate there was not an opportunity of realizing much profit from the rate of interest earned being higher than that assumed in calculating the premiums. This, the first valuation of the Fund, proves conclusively that the guaranteed annuities will in the future be materially increased out of profits." As this is the first quinquennial valuation, and many of the policy-holders have joined within the period under review, the Council have determined that the bonus additions will only be shown in the case of those policies which have been in force for the full period of at least four of the five years to which the first valuation relates. In he case of all other policies, although they have been credited with their proportionate share of the funds available, its precise amount will not be declared until after the next valuation.

The results of the quinquennial valuation must prove highly satisfactory to nurses, and this satisfaction will, no doubt, be increased by the following extract from the report of the Consulting Actuary:—"The Council and all members are to be congratulated on the very prosperous condition of the Pension Fund, disclosed by the valuation, and of the indications afforded by the large increases, which will be possible in the future, in the annuities of those nurses who do not withdraw from membership." Statements have been prepared under the fifth and sixth Schedules of the Life Assurance Acts, 1870, of the liabilities under the annuity and the sickness policies of the Fund, being the valuation as at December 31st, 1892, by the Consulting Actuary, Mr. George King. The statement is open to the inspection of policy-holders at the office, where it may be seen by them on personal application.

During the year the Council have had much pleasure in tendering their thanks to Dr. Potter for his generosity in presenting to the Donation Bonus Fund the amount of the

The Royal National Pension Fund for Nurses—*continued.*

royalties which he received from the publishers on account of his monograph, entitled, "Ministering Women," which contains a history of the Fund.

The Junius S. Morgan Benevolent Fund.—The work of this Fund has been so important during the past year, and has accomplished so much for the policy-holders, that the Council have thought it best to publish in full the report prepared by its committee. [It will be published in our next issue.] The Council beg to tender their most grateful thanks to Lady Rothschild, Miss Pritchard, the able Honorary Secretary, and the other ladies of the committee, believing as they do that the work carried on under their control is of great value and importance.

The Articles of Association provide that not more than eight representatives of the annuitants and policy-holders of the society may be added to the Council. The Council have, therefore, nominated the following ladies for election by the annuitants and the policyholders: Miss C. Davidson, Brompton Hospital for Consumption, Brompton; Miss E. Fisher, General Infirmary, Leeds; Miss L. M. Gordon, St. Thomas's Hospital; Miss K. H. Monk, King's College Hospital; Miss A. Ross, Victoria Infirmary, Glasgow; Miss E. Vincent, St. Marylebone Infirmary; Miss H. Wedgwood, Royal Free Hospital, member of the Council R.B.N.A.; and Miss A. Wood, Nurses' Co-operation, 8, New Cavendish Street, W.

There have been no changes in the permanent staff during the year, but the efficiency of the secretary, Mr. Louis H. M. Dick, and his assistants, will be gratefully recognised, when it is stated that the expenses of management, notwithstanding the large increase in the business of the Fund, are in fact less than they were during each of the two previous years.—By order of the Council, Louis H. M. Dick, Secretary.

Mr. W. H. Burns' Address.

The Chairman said: In presenting the seventh annual report there is little left for your chairman to say except to congratulate you upon the safe and steady progress which your Fund is making. Starting with a donation of £20,000, made by four public-spirited merchants of the City of London, we have already accumulated a sum of nearly £170,000, and we have an annual budget from nurses' payments and invested moneys of over £30,000.

We have traversed the period of tentative progress, and can now be ranked as a permanent and established institution, supported not only by the kindness of the general public, but, better still, reposing on the active co-operation of those we were formed to assist, and contributing not only to their material prosperity, but to the formation of habits of thrift most beneficial, in every way, to the nurses themselves.

The principal event with which this year has been marked for our society has been the preparing of our quinquennial valuation, a work which has been performed with the greatest care and devoted attention of our actuary, Mr. George King. You will see from the report that in every branch of our business we have obtained some profit, and while, though being divided over nearly 3,000 nurses, the individual result to each has not been very large. This is only the beginning of the benefit which our mutual and co-operative system will produce.

The early years out of the five years' period with which we have dealt could not realise much profit, as the difference of about 1 per cent. between the rate at which our policies are calculated and the rate of interest we have obtained has only been productive of any very important sum within the last two years, when a larger capital was employed in the business.

The report fully sets out the lines upon which the Council have proceeded in making the division of profits on the Donation Bonus Fund under the quinquennial valuation. The sum of £5,000 was given, with the stipulation that its benefits were to be confined to the first thousand nurses. This has been done with their share of the profit on the said £5,000. The remainder of the Fund applies to all the nurses, and your Council have decided to apply three-quarters of the profits shown in augmenting the pension of each nurse *pro rata* to the amount of their payments to the Fund. For the more perfect understanding of the system pursued I now give a copy of the minute of the Council under which the actuary has made his division.

"That £5,000 of the capital of the Donation Bonus Fund, together with the whole of the accumulated interest on the said £5,000, be divided equally among those of the first thousand nurses who are still members of the Fund. These equal cash appropriations to be turned into annuities of corresponding kinds to the annuities already held by these nurses. This special fund of £5,000 and interest to be kept always separate as a Tontine Fund, and annuities provided by it to be cancelled if the beneficiaries from any cause cease to belong to the Fund, and the values of such cancelled annuities to be applied at each valuation to increase the annuities of those of the first thousand nurses who still remain with the Fund."

"Three-fourths of the remaining interest in the Donation Bonus Fund to be carried to the Annuity Fund, and to be divided among all nurses holding annuities, in proportion to the payments received from them accumulated at three per cent. compound interest; these cash appropriations to be turned into annuities of corresponding kinds to the annuities already held by the nurses."

The remaining quarter of the profits in addition to the general profits of the business under annuity and sickness contracts remains on hand unappropriated. The Fund, therefore, starts the second quinquennial period with over £2,000 of accumulated profits on hand. Your Council thought—and the distribution is left entirely to their discretion—that it would be to the interests of the nurses not to divide up everything at once, as our business was a new one, and we had much to learn both as regards the rate of mortality of nurses and their liability to sickness, and therefore it was prudent to keep something in hand to meet the possible contingencies of the future.

With regard to the investments of the Fund, you will be glad to learn that they are all sound, and, I believe, satisfactory.

During the past year the sickness branch has shown more adverse results than in the past, as we paid away in sickness allowance nearly 50 per cent. more than in 1892, our sick pay being £576 7s. 6d., against £737 0s. 4d. received, or say 78 per cent. of the year's premiums. During the period from January, 1889, to December 31st, 1892, our actuary reports that the sick pay actually drawn was £837 0s. 7d., and had our experience corresponded with the tables of the Manchester Unity of Oddfellows, on which the rates of premium of the Fund were based, there would have been drawn £857 10s.

It is satisfactory to note that the rate has so far proved protective to the Fund and goes to show that the risk of sickness in nurses does not exceed that of the average community, but it must be remembered that in these early days many of the nurses have only recently passed the medical examination, and it will be most necessary for the Council to admit only healthy and strong nurses to the benefits of the sickness assurance.

Of the nurses' policies 116 have been surrendered and 106 nurses have withdrawn from the Fund; of these 66 have done so through death, marriage, or giving up nursing, leaving 40 for various reasons, such as ill-health, inability to keep up payments, going abroad, &c.

I regret to state that owing to the press of other business Mr. Slaughter has relinquished the office of honerary solicitor to the Fund, and Mr. Percival A. Nairne has been appointed solicitor in his place. The thanks of the members and annuitants are due to Mr. Slaughter for the honorary services which he so kindly rendered from the creation of the fund.

I cannot close these few remarks without a tribute of gratitude to the devoted attention and labour of Mr. Dick, our secretary, of Mr. Pocock, the assistant secretary, and the clerical staff. Some idea of their work can be formed when I state that over 30,000 letters have come in and out of the office during the year, and that we estimate that each policy adds seven letters and over twelve book entries per annum. Notwithstanding the natural increase of business, the work has been thoroughly carried out, and new books and registers have been opened, making the labour of future years a much easier matter. Deducting the special payment of £263 14s. 11d. for the expense of the quinquennial valuation, the amount of working expenses was £1,428 9s. 3d. for 1893, against £1,412 9s. 10d. for the preceding year.

The Chairman then moved the adoption of the report and the accounts, which was seconded by Mr. Burdett and carried. The retiring members of Council were unanimously re-elected.

The Chairman said the next business is the re-election of auditor. Mr. Frederic Whinney (of the firm of Messrs. Whinney, Hurbatt, and Smith, chartered accountants,) again offers himself for that post. I move that Mr. Whinney be appointed auditor. Mr. Arthur H. A. Morton seconded, and it was passed unanimously.

Annuitants and Policy-holders' Representatives.

The Chairman said the scrutineers gave the following report of the ballot: L. W. Gordon, 1,141 votes; E. Vincent, 1,134; R. H. Monk, 1,127; E. Fisher, 1,125; A. Wood, 1,120; C. Davidson, 1,118; A. Ross, 1,115; H. Wedgwood, 1,108. For some other names votes were recorded, ranging in number from 15 votes to 1. The scrutineers mentioned that 41 cards were returned bearing no signature. He now proposed a vote of thanks to the scrutineers, Mr. H. M. Macpherson and Dr. Potter for their services. This resolution was adopted.

Mr. Burdett: I rise to propose a vote of thanks to the Chairman. It has been my privilege to do so every year since the fund was established. I shall express my own feelings, and echo the sentiments of my colleagues on the Council, if I say that every year, as I stand up to move this vote of thanks, I am more and more impressed with the importance of the trust placed in my hands. Mr. Walter H. Burns, the more we know of him, the more we not only like but value him. He is always working for us; and only in the last fortnight we have had an instance of his generous interest in the Fund, and his determination, at whatever cost, that this Fund shall prosper and increase. To us he is a dear friend and colleague; to you he is the chairman of the Pension Fund. You may take it from us, and we are united in this, that everybody must feel that to Mr. Burns our most grateful and earnest thanks are due for having, in the first instance, undertaken the duties of chairman, and for having with such unselfish devotion performed the duties for so many years in succession.

Mr. G. King: I may be allowed to second this vote of thanks. It does not require any more to be said. The Fund itself is a sufficient monument of the skill with which it is managed, and of the chairman, who has most to do with the investments. A Fund like this depends much on the soundness of the investments, and in these days of difficulties to be able to report that all the investments are sound, is about the highest credit that could be given to any board of directors. It was a great pleasure to me to make the actuarial valuation. It was by no means an easy thing to do. It was quite a unique experience to value such a Fund as this. But it shows how safe the Fund itself is, and what a pleasure it is to all who have had anything to do with it. This motion was carried by acclamation.

The Chairman: Mr. Burdett, Mr. King, ladies and gentlemen, I am extremely grateful to you for the more than kind way in which allusion has been made to any services I have been able to render the Fund. I first entered upon my connection with this fund at the request of my late father-in-law, who took a deep interest in it, and I did so with the desire to forward its interests, and contribute what I could to the helping of the nurses. The longer I remain at the helm and see the working of the Fund, the more personal my feelings become in the matter; and now I feel it is a very great privilege to have been allowed to occupy the position I have had in the starting and establishing of a Fund which I think will be found by my successors in a perfectly sound and workable condition. Mr. Burdett has said so many kind things of myself personally, I scarcely know how to thank him sufficiently. One thing he said was that it was his duty or pleasure to rise year by year to propose this resolution; and I can say that I hope that for many years he will fill the same function, even if not towards me, yet towards other presidents. ("To you," "to you," and cheers.) I value his co-operation, I need not say. Without Mr. Burdett's energy in founding this institution—teaching us all our work, and helping us to do it—we should not only be not prosperous, but we should not be here at all.

This concluded the proceedings of the annual meeting of 1894.

### Guy's Hospital Trained Nurses' Institute.

Rules and Regulations.

A most satisfactory report has been issued by the Treasurer of the Guy's Hospital Trained Nurses' Institution, the number of nurses having risen from 62 to 82 during the last twelve months. The Lady Superintendent, Miss Swift, was appointed early in 1893 as successor to Miss Nott-Bower, who then became Matron of Guy's Hospital. The rules for the private nurses are simple and sensible ones: "The nurse shall faithfully tend and minister to the sick person, behaving herself with tact, gentleness, and discretion. She shall report any symptoms she may observe to the medical attendant, and shall carry out his directions to the best of her ability. She shall abstain from expressing an opinion upon diagnosis or treatment as beyond the province of a nurse, referring inquiries upon such subjects to the practitioner in charge of the case. . . . She shall use her best endeavours to work harmoniously with the relatives of the patient and the rest of the household. She shall carefully avoid giving unnecessary trouble, and, when required, she shall keep her own and the patient's room clean and tidy. . . . It is expected that the nurse should be treated with kindness and consideration by the patient and his family, and that regard should be had to her health. . . . The nurse's food should be plain and good, and her meals served separately, neither in the sick room nor with the servants. . . . She must wear her nurse's dress when on duty in the house, and at all times out of doors."

Maternity Nurses.

The two maternity nurses maintained by Guy's Hospital have paid 3,193 visits during the year in those districts embraced by the lying-in charity. Last year an appeal was made for the Maternity Samaritan Fund, and this being liberally responded to, clothing, food, coals, and other necessaries have been provided for destitute mothers. Presents of linen and old clothes are constantly required, and gladly acknowledged by the Lady Superintendent, 14, St. Thomas's Street, E.C.

Nurses Fees and Pensions—A Model Scheme.

A nurse whilst she is a probationer receives £12 for the first, and £18 for each of the two succeeding years. At the completion of her training she can join the permanent nursing staff at £25 for the first, £30 for the second, £35 for the third, and £40 for the fourth year. In addition to her salary the institution takes out for her a policy in the Royal National Pension Fund, and these policies have been altered so as to secure to each a yearly pension of £11 5s., instead of £7 10s., as formerly, thus placing the institution and the hospital nurses on an equal footing.

It must also be borne in mind when considering the position of those attached to the private nursing staff of the hospital that uniform, washing, and medical attendance are provided; also board and lodging between cases, and skilled nursing and attendance in case of illness. In comparing the relative positions of nurses "on their own account" and those at hospitals, these advantages are apt to be lost sight of, or at any rate to have less appreciation than they deserve.

The bonus scheme which has been introduced at Guy's Hospital provides for the division of the surplus profits "amongst those nurses who have been on the staff of the institution for four full years subsequently to the completion of three years as probationers . . . is invested for their benefit with the Royal National Pension Fund for Nurses for the purchase of pensions to begin at the age of fifty-five. . . . Should a nurse die before attaining the age at which her pension falls due, the money standing to her credit will be paid out of the Fund to any person whom she may nominate, or if she should become permanently incapacitated from following her avocation as a nurse the money may be applied towards her maintenance or for her benefit in such manner as may be thought expedient. . . At the age of fifty, when the bonuses cease . . . she becomes entitled to her own pension of £7 10s., plus the pension of £11s. 5s., for which the institution has paid the premiums. Dr. Perry may certainly be congratulated on the promotion of a scheme which is so thoroughly advantageous to each individual nurse.

### What a Nurse Should Read.

By A. E. I.

It so much depends on the individual nurse, that in giving advice on this subject one immediately realises the fact that the nursing world comprises "all sorts and conditions of women." In these days, when elementary physiology and anatomy are taught in most schools, many of the younger nurses commence their training well grounded in the A B C of the work which lies before them. Again, many older women who take up nursing have already studied quite deeply advanced medical works, but would not be able to say decidedly if the "femur" was in the arm or leg. But I will presume that the majority have commenced their training with either no knowledge, or with the merest smattering of scientific lore, but with an intense love for their work, and a desire to gain knowledge. To these, therefore, I offer the following remarks, trusting that they will be of use, and that all those now beginning their training may find each step of the way full of interest, and, if never before, they may now appreciate the lines in the "Village Blacksmith"—

> "Something attempted, something done,
> Has earned a night's repose."

"Read, mark, learn, and inwardly digest," is the bsst kind of motto which could possibly be taken by anyone wishing to read with understanding. Reading at lightning speed, or glancing first at one book and then at another, only gives a confused jumble of ideas and words, and the knowledge (if any) thus gained is useless. Read slowly, read thoughtfully, and so make the knowledge your own. Until you know a thing so well that you could explain it to another, you have not thoroughly mastered it yourself. If you have a friend willing to be victimised by listening to your newly acquired knowledge, try to write it down; if you commit your knowledge to paper it enables you to judge how much you really know.

To have a general idea of what is meant thereby whilst you are actually reading will not help you afterwards. Accuracy is of the greatest importance, more especially in the beginning, for a wrong impression, once formed, is difficult to get rid of.

A note-book is essential. Any word, expression, order, instrument, &c., that you hear named, or see, but don't understand, should be noted down at once and not scratched off the note-book, until in some way you have gained the required information. Sometimes you can ask the sister or charge nurse under whom you are working; but new probationers have not always the opportunity even if they have the courage to do this, and if a fellow probationer cannot tell you, you may have to hunt up the knowledge from books. Never rest until you know; the difficulty you experience in finding out what you require will impress the fact for ever on your memory. And with regard to asking questions, a little tact may often obtain the required answer. Do not propound your puzzles at all times, and especially during the busiest parts of the day. I remember a probationer who gained for herself the unenviable name "The note of Interrogation," because a question was for ever on her lips. When hurrying past her on the most urgent duty a nurse might be inconveniently arrested by the needless query, "What is that for?" or "Why are you in a hurry?"

Write down one new fact every day, for one fact a day makes 365 learnt in a year. Later on you will be able to arrange them in order and make for yourself an invaluable private book of reference. Read at all times and in all places, not necessarily from books alone. Printed words may make your eyes dim and your head ache, and then no sense is communicated to the brain. But in your wards as you go about your daily work you have a book open before you that it only requires attention to comprehend.

When I see a probationer on night duty bending over a book with small print, a bad light possibly hurting her eyes, and then observe her rise with an impatient jerk to attend to some patient's want, I am forcibly reminded of the tourist who, whilst passing through some of the most beautiful scenery in the world, kept his eyes bent on a guide-book, reading someone else's description of the scene. Guide-books are useful, but they do not take the place of observation. On night duty, even in the quietest ward, there is much to be learnt. The position in which the patients lie, the kind of sleep, the breathing of each one, are amongst the things to be noted. To remark if all the patients suffering from one complaint breathe in the same way, lie in the same position, &c. Afterwards you can easily compare your experience with your "guide-book," and see in how far they agree with each other. Then, again, the opportunities of study when on duty with a "special case" are unlimited. Keep an accurate report, and, besides the mere facts that you write down, take notes for yourself of the treatment, and of any special feature in the case that strikes you as new; then, when you have other patients suffering from the same kind of disease, you will be able to make interesting comparisons. No knowledge gained from books alone can ever take the place of what you see and find out for yourself. I lay special stress on this reading at first hand because it has been my experience that many nurses neglect to take advantage of all the grand opportunities in their hospital training, forgetting that they will rarely recur again.

But as books are necessary, I should recommend a new probationer to get Florence Nightingale's "Notes on Nursing: (First Aid to the Injured, and Home Nursing and Hygiene)," published by the St. John's Ambulance Association; also a good medical dictionary—the dictionary to be kept in her room; Miss Lückes' "Lectures on Nursing," and Huxley's "Elementary Physiology." I am aware that my selection of books is very simple, but I think as a rule they are quite advanced enough for the average probationer; if she masters all the knowledge contained in them she will do well for the first year, and after that the special line of nursing she wishes to follow will guide her in her choice of books. It is not unusual to see a new probationer puzzling her brain over some book more fitted for a medical student passing his final examination. Even if she understands, the knowledge thus gained is of little use to a nurse.

There are many delightful books on nursing, both English and American, which I would recommend to those who have passed through their first year of probation, and the catalogue of the Scientific Press, 428, Strand, is to me a really wonderful revelation of the number of books existing for the special instruction of myself and my fellow nurses.

### Novelties for Nurses.

Mr. Egerton Burnet, Wellington, Somerset, is always to the fore with useful and attractive dress materials for nurses, and the packet of serges and washing fabrics which has just reached us shows no exception to the usual high standard of excellence. The serges are of useful colours, and firm and strong in texture, giving promise of good wear, with a wide range of prices, coming within the reach of all. The "cloaking serges" for uniform cloaks are especially good. There are some pretty washing materials in all colours, striped, checked, and plain, but where all are good it is hardly possible to point out any one of these many alluring patterns as being better than another. Mr. Burnet's new price list for 1893-4, gives comprehensive particulars of the materials supplied from the "Jubilee Royal Serge Warehouse, Wellington."

☞ For Everybody's Opinion, Reading to the Sick, and Book World for Women and Nurses, see page ccxlix. et seq.

### "Nervous Heredity in Villages."

M. Zola, with his volume entitled "Dr. Pascal," has at length completed the long series of novels generally known as "Les Rougons-Macquart," a score or so of volumes put forth at intervals ever since the French war. This last "chapter" of the series is admirably translated by Mr. Vizetelly; in fact, the book is one of the best pieces of translation which has ever been done. Modern French is not easy to transfer into modern English without keeping painfully before the reader's mind the fact that it is French, and M. Zola's French is not the easiest; but the translator, from his habitual knowledge of both languages, and his consummate touch with Continental thought, has performed his task to perfection. He has omitted little, for there was less which called for omission than we are accustomed to find in many of this author's volumes, but the discarded matter has done no hurt to the continuity of story or idea.

Every physician will find much food for thought in "Dr. Pascal," and, we venture to think, no class of medical men more than country practitioners. As is well known, the "Rougons-Macquart" series of novels is an attempt to give the life history, and prominently the psychological family history, of a Provençal family, affected by hereditary neurosis. The volumes are scenes in every phase of French life, the principal actors being members of one family, all, or nearly all, showing some hereditary cerebral taint, while many of the books introduce, not always to their advantage, a minute picture of the details of different professions and trades, which must have cost a large outlay of patient research. Zola's history of this neurotic Rougon family, with its illegitimate Macquart branch, is a perfectly true picture, showing five generations descended from one neurotic ancestor, in which ill-defined psychoses appear first, to be succeeded by more definite psychoses, and in a further generation by nerve-scleroses, with the final extinction of the family, except where some lines have had an admixture of healthy country blood. The psychical side of the neuroses of course introduces the dramatic interest, an interest which we may describe as fanned by the hot-air blast of "realism"; the moral mania of the family finds its culminating examples in such fearful portraits as Coupeau, the dipsomaniac in "L'Assomoir," his daughter Nana, who, like "Cora Pearl," was a specimen of the fungi grown on the dung-heap of the Paris of thirty years ago; and Jaques Lantier (in "La Bête Humaine"), a homicidal maniac of the type of "Jack the Ripper."

We review M. Zola's scheme thus in order to point out how much interest and profit medical men may derive from a similar careful and intimate observation of real family histories; and no man has such a good opportunity as the country practitioner in districts where populations are more stationary than in towns. We all know how impossible it is to obtain correct family histories second-hand from our patients or their relations; and this applies particularly to neuroses. Not only are quite intelligent men and women usually very ignorant of their own pathological family histories, but they are also very shy of acknowledging even a definite neurosis in their forbears or relations, while they are also quite ignorant that many mental traits and failings (such as alcoholism) are in reality evidences of a neurosis at all. It is only the cultivated physician who knows personally numerous members of a family in several generations who can piece together such a network of evidence; and this can only happen in villages and country towns.

It is a great error to suppose that neuroses are chiefly the portion of dwellers in towns; every tiny village in England can show a list of imbeciles, dipsomaniacs, epileptics, hysterics, and other neurotics, if the poor-law medical officer were applied to. Not only so, but there is good evidence from America that neuroses are most prevalent where a quiet country life is being rapidly converted, by the construction of new railways, or the growth of neighbouring large centres, into a more active, more competitive existence. The nervous system, unaccustomed by heredity to rapid changes, is more prone to resent such disturbance, and show its resentment by a want of equilibrium, than one which has in a few generations gradually learnt to adapt itself to the bustle of modern civilisation.

Furthermore, the country doctor has the opportunity of seeing how such trophic neuroses as diabetes, ostroarthritis, gout, are related by heredity with recognised nervous diseases.

An important factor is at work in country villages tending to produce hereditary neurosis which is absent in towns. Every village contains feeble-minded young men and women, who are yet not quite imbecile, the products of ill-fed, ill-educated, neurotic families. Every country vicar knows how readily these youths and maidens marry each other; few months pass but he has to publish banns of marriage which he would fain forbid, but has no power to do so. These half-witted girls and youths mate each other because they have no chance of securing other mates, and being on the verge of chronic starvation, they produce an ill-fed, sickly, neurotic brood, fortunate if it all dies of convulsions in dentition.

### Queen's Nurses at Worthing.

The annual meeting of the Worthing District Nursing Association, affiliated with the Queen's Jubilee Institute, took place on the 10th inst, and was very largely attended. The splendid work done by the permanent and also by the extra nurses during the typhoid epidemic was cordially acknowledged. Miss Peter, Inspector of the Institute, had reported most favourably of the nursing at Worthing, and her good opinion has been heartily endorsed by the sufferers who have benefited by the professional attendances of Miss Burkitt and Miss Buckle. Amongst other speakers at the annual meeting Miss Hughes of the Central Training Home, Bloomsbury, gave a most interesting address, detailing the growth of the nursing movement, and explaining the necessity for Queen's Nurses being adequately trained in district work as well as undergoing a full course of hospital training. Miss Louisa Twining also spoke of the progress which had been made by trained nursing in various departments, and remarked that the Worthing Guardians had been the first to make a payment for the services of nurses—an example which might be advantageously followed by other Boards. Sir Henry Fletcher, who presided, and Dr. Goldsmith also spoke cordially of the valuable work accomplished by the district nurses in Worthing.

### Where to Go.

The Imperial Institute.—During the first week in May the Annual Amateur Art Exhibition will be held on behalf of the Parochial Mission Women's Fund, the East London Nursing Society, and the East-end Mothers' Home. Intending exhibitors are asked to communicate with the Hon. Sec., the Hon. Mrs. Stuart Wortley, 16, Clarges Street, Piccadilly.

### Wants and Workers.

Will anyone kindly inform me of a Home where a blind man can be taught an industry; and also of one where a girl with a crippled arm could be received?—*M. E. H.*

### Everybody's Opinion.

Correspondence on all subjects is invited, but we cannot in any way be responsible for the opinions expressed by our correspondents. No communications can be entertained if the name and address of the correspondent is not given, or unless one side of the paper only be written on.]

### MATERNAL FEELINGS IN DEMENTIA.

"R. G. W." writes: Reading Dr. Greenlees' article in The Hospital of February 24th reminds me of a case in which it seemed for a time as if maternal love had died out. Yet although a woman be prevented by disease from showing a mother's tender care, I cannot but think that it, to a certain extent, exists still—even if hidden behind a stolid demeanour. At any rate, it was so with the young woman in question. Her state is actually pictured by Dr. G.'s description of dementia — never speaking, perfectly apathetic, very obstinate, and taking no interest in anything. What was my surprise, being one day in the reception-room, to find a beautiful, manly little fellow of four or five hugging the patient with all his might saying "Oh, dear mamma, I do love you so! Do come back soon!" I took the opportunity to say, "Why, Mrs. S., I am sure you will do your best to get well now," and I praised and petted the little chap. I cannot say she ever made much progress towards recovery, but whenever I had a chance I used to remind her of her little son, and tell others about the beautiful boy, and though she never joined in the conversation, yet her eyes shone, and it evidently pleased her. And, strangely enough, obstinate and contrary as she had always been, I had afterwards but to suggest a thing, and before the words were hardly out of my mouth she would do it. She had not outwardly responded to the child's warm affection or answered his appeal to come home, yet, I believe, it was the hope of her heart. Could it be that an instinct told her her company was not at that time desirable for the child? It is in these cases that thoughtful sympathy can keep alive the germ of better feelings and fan the flickering flame of love and reason. How can anyone talk of dulness and monotony when such tasks as these await a mental nurse?

### "FIRST AID."

"T. B." writes: In the prize answer for February, published 10th inst., there is the following statement regarding the carrying of the stretcher: "The bearers must keep step in order to avoid any undue movement." If the writer of the article had had any practical experience of stretcher work, as a bearer or as a patient, she would know that keeping step causes a regular swing of the stretcher, which certainly must be characterised as undue movement. And in all authorised drill books, as of the M.S.C. and the St. John Ambulance Brigade (to which body I am proud to belong), there is the distinct instruction, "Bearers to keep broken step," the rule being for No. 1 bearer, at the foot, to step off with the left foot, and No. 3 bearer at the head, with the right. In other respects the article shows experience and knowledge, but this is rather an important point.

### Presentation.

The Committee of the Bradford Incorporated Nurses' Institution presented Miss Bartlett with a handsome diamond ring, and the private nurses with a gold bar brooch and a travelling clock, on her departure for her new work of superintendence in a London Home Hospital.

### For Reading to the Sick.

### CHRIST'S LOVE IN THE PASSION.

**Motto.**

Shun not suffering shame or loss,
Learn of Him to bear the Cross.

—*Montgomery.*

**Verses.**

By anguish that made pale the sun,
I hear him charge His Saints, that none
Among His creatures anywhere
Blaspheme against Him with despair—
However darkly days go on.
Take from my head the thorn wreath brown—
No mortal grief deserves that crown!
O Supreme Love! Chief Misery!
The sharp regalia are for Thee,
Whose days eternally go on!

—*E. B. Browning.*

With all His sufferings full in view,
And woes to us unknown,
Forth to the task His spirit flew;
'Twas love that urged Him on. —*Cowper.*

**Reading.**

"When I Survey the Wondrous Cross," we sing. Wherein consists its wonders?

1. It is wonderful, because it tells of the greatness of the love of Christ for every created soul.

Inscribed upon the Cross we see
In shining letters, "God is Love."

The very form of the crucified Lord speaks to us in a mystery of His great love; the arms stretched out as though He would embrace the world, the head bent low to receive back each returning penitent, the hands and feet pierced, that in those blessed wounds wearied, heavy-laden contrite souls may hide, as it is written, "Thou art a place to hide me in;" "Scarcely for a righteous man will one die, yet, peradventure, for a good man! some even dare to die. But God commendeth His love towards us, in that while we were yet sinners. Christ died for us. . . ."

2. It is a Cross of wonder because of the miracles of converting grace which it has wrought. "The preaching of the Cross is to them that perish foolishness"; "but unto them which are saved it is the power of God." The old story of the Cross dyed purple with the Precious Blood, how often has it changed the current of a man's life, as it seemed quite suddenly. Wherever the Passion of our Lord is preached some sinners will be melted to penitence, tears will well over from strong men's hearts, lives long dedicated to selfishness and sin will be transformed, renewed, made glorious by the miraculous virtue of Calvary. "They overcame him by the Blood of the Lamb." Nor is it sin alone, but sorrow too, is vanquished by the Cross. Each day agonised sufferers endure untold pains without a murmur, because they have learnt how to suffer by meditation on the saving Cross. "My flesh and my heart faileth," they cry, "but God is the strength of my heart and my portion for ever."—*T. Birkett Dover.*

We know ourselves that the more we do for anyone, the more we suffer for anyone, the more we love them; the mother loves that child most whose delicacy has made her sacrifice her rest for it, or spend a fortune on its treatment; and He who loved us much at the beginning, when He was born for us, loved us more when He had died for us. —*Canon H. W. Burrows.*

O unexampled love!
Love nowhere to be found less than Divine!

—*Milton.*

### The Book World for Women and Nurses.

[We invite Correspondence, Criticism, Enquiries, and Notes on Books likely to interest Women and Nurses. Address, Editor, THE HOSPITAL (Nurses' Book World), 428, Strand, W.C.]

THE CURB OF HONOUR. By M. BETHAM-EDWARDS. (London: Adam and Charles Black, 1893.)

Does it chance to be the case with Miss Betham-Edwards that the English of ordinary everyday use, is insufficient for the needs of her soul's expression? or is it with the wish to substitute the older forms of expression for the newer that we find so startling an array of uncommon words in this latest addition to the novelist's list of works? In speaking of the beauty of her heroine the authoress describes her with a "Vesper" charm, "inconspicuous" though it was at first, and equally so, we are afraid, at the last to all but the mind which conceived the character. "An innate misshapenness," also characterized the hero, of whose face the writer says, "Most noteworthy about this countenance, 'unhandsome,' yet at times how beautiful, was its fixity and strange look of aloofness from actualities." Another personage in the book is described by Miss Edwards with equal disregard to conventional language, Miss Tait, the authoress remarks—"regarded virtue as an empty shadow unless turned into self-flagellation." After three hundred pages the story winds up in the manner one foresaw from the commencement it would do, and the hero's "up buoyance" of spirit and "anticipatory endurance" are rewarded by the hand of the lady above-mentioned, whose view of virtue was so forcibly described by Miss Edwards' pen.

HEALTH RESORTS FOR TROPICAL INVALIDS. By Sir W. J. MOORE. (J. and A. Churchill, London.)

This is a motherly sort of book. A quasi-scientific, quasi-popular digest of guide books and works innumerable on climatology and travel. The author claims that there is no one work affording the information to be found in the pages of this work. We do not deny it. For a work of this kind to be of material use there should at least be some attempt at a rational classification either according to climate or according to disease. The author, however, has recourse to a classification of health resorts according to their alphabetical order. He divides his work into sections treating of (1) Hill Stations; (2) Tropical Marine Sanitaria; (3) Health Resorts Abroad; (4) Health Resorts at Home. Each section is prefaced by a few generalisations to assist the reader in a choice of a suitable locality. Remarks of value will be found on pages 80-83, where he treats of the respective merits of foreign health resorts for the various forms of disease acquired in India.

HEALTH AND THE VARIOUS METHODS OF CURE. By J. H. RAUSSE. (London: L. N. Fowler and Co.)

This is a small work, which in about 100 pages disposes of the whole question of therapeutics. It does so in the following manner. All drugs are poisonous, and therefore are to be avoided. Allopathy is rather worse than homœopathy, since it prescribes poisons in larger doses. "It is easy enough," says the author "to explain how Hahnemann bethought himself of his decillion particles. Convinced of the harm the former large doses of poisons did, he tried to make them smaller and smaller, thus succeeding better and better the less poison he gave—so natural. For so much more freely the organism itself could develop its healing power." The author exhibits a lamentable ignorance of the uses of drugs, and a commendable knowledge of the classics and mythology, from which he culls a perfect bouquet of flowery quotations; trimming and garnishing them, until by specious argument, and the suppression of the truth, they apparently prove his point. If a work of this kind be put in the hands of the ignorant, and accepted seriously, there is no estimating the mischief it may cause. It is obviously written for a purpose, and that is to advertise a method of cure, and should deceive no one. We do not propose to aid or abet the purpose in describing that method.

A GENTLEMAN OF FRANCE. By STANLEY J. WEYMAN. New Edition. (London: Longmans, Green, and Co. 1894.)

The evident care and painstaking as to elaboration of detail in working out the plot of this book has reaped for its author a fair reward. "A Gentleman of France" has just passed into a new edition, in a one-volume form this time. The Sieur de Marsac, who tells the story himself, does so in a natural and a taking manner, and gives us a good picture of the times in which it is intended we should fancy ourselves at his bidding. Indeed, the hero succeeds in touching up one's memory in an effective manner about the France of the years immediately succeeding the St. Bartholomew. But the book is by no means purely an historical one; a romance in deftly intertwined and worked in, with perhaps a greater consistency and probability of truth than is the case with the more impassioned novels of the *fin de siècle* order.

CHARACTER AND FORTUNE REVEALED. An A B C Guide to Palmistry. PAUL BELLO. (David Stott, London.)

This is a small but complete manual of palmistry. Although we live in such a matter-of-fact age, palmistry has never found a larger number of those who show interest in it, though these would not always go so far as to call themselves believers. Palmistry never fails to excite a certain amount of attention where it is practised, and is always a popular form of entertainment. There is no theme so interesting to ourselves as ourselves. To those who take the subject up, on whatever grounds, this little book cannot fail to give satisfaction. The introduction gives the general plan or method of dividing the subject. Then follows a vocabulary of terms, and afterwards the separate study of typical hands, with the many exceptions and deviations minutely pointed out. Not a few will be daunted by the immense amount of modifications of the rules laid down, which have to be mastered before the student is proficient. To those who have already studied the subject, the A B C will open fresh fields of investigation, and prove a welcome addition to their store of knowledge.

